# Fibonacci statistical convergence and Korovkin type approximation theorems

**DOI:** 10.1186/s13660-017-1503-z

**Published:** 2017-09-19

**Authors:** Murat Kirişci, Ali Karaisa

**Affiliations:** 1 Department of Mathematical Education, Hasan Ali Yücel Education Faculty, Istanbul University, Vefa, Fatih, Istanbul, 34470 Turkey; 2 Department of Mathematica-Computer Science, Necmettin Erbakan University, Meram Campus, Meram, Konya, 42090 Turkey

**Keywords:** 11B39, 41A10, 41A25, 41A36, 40A30, 40G15, Korovkin type approximation theorems, statistical convergence, Fibonacci numbers, Fibonacci matrix, positive linear operator, density

## Abstract

The purpose of this paper is twofold. First, the definition of new statistical convergence with Fibonacci sequence is given and some fundamental properties of statistical convergence are examined. Second, we provide various approximation results concerning the classical Korovkin theorem via Fibonacci type statistical convergence.

## Introduction

### Densities and statistical convergence

Let *A* be a subset of positive integers. We consider the interval $[1,n]$ and select an integer in this interval, randomly. Then the ratio of the number of elements of *A* in $[1,n]$ to the total number of elements in $[1,n]$ belongs to *A*, probably. For $n\rightarrow \infty$, if this probability exists, that is, this probability tends to some limit, then this limit is used as the asymptotic density of the set *A*. Let us mention that the asymptotic density is a kind of probability of choosing a number from the set *A*.

Now, we give some definitions and properties of asymptotic density.

The set of positive integers will be denoted by $\mathbb{Z^{+}}$. Let *A* and *B* be subsets of $\mathbb{Z}^{+}$. If the symmetric difference $A\Delta B$ is finite, then we can say *A* is asymptotically equal to *B* and denote $A\sim B$. Freedman and Sember introduced the concept of a lower asymptotic density and defined the concept of convergence in density, in [1].

#### Definition 1.1

[1]

Let *f* be a function defined for all sets of natural numbers which takes values in the interval $[0,1]$. Then the function *f* is said to be a lower asymptotic density if the following conditions hold:

i. $f(A)=f(B)$ if $A\sim B$;
ii. $f(A)+f(B)\leq f(A\cup B)$ if $A\cap B=\emptyset$;
iii. $f(A)+f(B)\leq1+ f(A\cap B)$ for all *A*;
iv. $f(\mathbb{Z^{+}})=1$.

We can define the upper density based on the definition of lower density as follows.

Let *f* be any density. Then, for any set of natural numbers *A*, the function *f̅* is said to be upper density associated with *f* if $\overline{f}(A)=1-f(\mathbb{Z}^{+} \backslash A)$.

Consider the set $A\subset\mathbb{Z}^{+}$. If $f(A)=\overline{f}(A)$, then we can say that the set *A* has natural density with respect to *f*. The term asymptotic density is often used for the function

$$d(A)=\liminf_{n\rightarrow\infty}\frac{A(n)}{n}, $$

where $A\subset\mathbb{N}$ and $A(n)=\sum_{a\leq n, a\in A}1$. Also the natural density of *A* is given by $d(A)=\lim_{n}n^{-1}\vert A(n)\vert $, where $\vert A(n)\vert $ denotes the number of elements in $A(n)$.

The study of statistical convergence was initiated by Fast [2]. Schoenberg [3] studied statistical convergence as a summability method and listed some of the elementary properties of statistical convergence. Both of these mathematicians mentioned that if a bounded sequence is statistically convergent to *L*, then it is Cesàro summable to *L*. Statistical convergence also arises as an example of ‘convergence in density’ as introduced by Buck [4]. In [5], Zygmund called this concept ‘almost convergence’ and established the relation between statistical convergence and strong summability. The idea of statistical convergence has been studied in different branches of mathematics such as number theory [6], trigonometric series [5], summability theory [1], measure theory [7] and Hausdorff locally convex topological vector spaces [8]. The concept of *αβ*-statistical convergence was introduced and studied by Aktuǧlu [9]. In [10], Karakaya and Karaisa extended the concept of *αβ*-statistical convergence. Also, they introduced the concept of weighted *αβ*-statistical convergence of order *γ*, weighted *αβ*-summability of order *γ* and strongly weighted *αβ*-summable sequences of order *γ* in [10]. In [11], Braha gave a new weighted equi-statistical convergence and proved the Korovkin type theorems using the new definition.

#### Definition 1.2

A real numbers sequence $x=(x_{k})$ is statistically convergent to *L* provided that for every $\varepsilon>0$ the set $\{n\in \mathbb{N}: \vert x_{n}-L\vert \geq\varepsilon\}$ has natural density zero. The set of all statistically convergent sequences is denoted by *S*. In this case, we write $S-\lim x=L$ or $x_{k}\rightarrow L(S)$.

#### Definition 1.3

[12]

The sequence $x=(x_{k})$ is statistically Cauchy sequence if for every $\varepsilon>0$ there is a positive integer $N=N(\varepsilon )$ such that

$$d \bigl( \bigl\{ n\in\mathbb{N}: \vert x_{n}-x_{N(\varepsilon)}\vert \geq\varepsilon \bigr\} \bigr) =0. $$

It can be seen from the definition that statistical convergence is a generalization of the usual notion of convergence that parallels the usual theory of convergence.

Fridy [12] introduced a new notation for facilitation: If $x=(x_{n})$ is a sequence that satisfies some property *P* for all *n* except a set of natural density zero, then we say that $x=(x_{n})$ satisfies *P* for ‘almost all *n*’, and we abbreviate ‘a.a.n’. In [12], Fridy proved the following theorem.

#### Theorem 1.4

*The following statements are equivalent*:

i. *x* *is a statistically convergent sequence*;
ii. *x* *is a statistically Cauchy sequence*;
iii. *x* *is a sequence for which there is a convergent sequence* *y* *such that* $x_{n}=y_{n}$ *for a*.*a*.*n*.

### Fibonacci numbers and Fibonacci matrix

The numbers in the bottom row are called *Fibonacci numbers*, and the number sequence

$$1,1,2,3,5,8,13,21,34,55,89,144,\ldots $$

is the *Fibonacci sequence* [13].

#### Definition 1.5

The Fibonacci numbers are a sequence of numbers $(f_{n})$ for $n=1,2,\ldots$ defined by the linear recurrence equation

$$f_{n}=f_{n-1}+f_{n-2}, \quad n\geq2. $$

From this definition, it means that the first two numbers in Fibonacci sequence are either 1 and 1 (or 0 and 1) depending on the chosen starting point of the sequence and all subsequent numbers is the sum of the previous two. That is, we can choose $f_{1}=f_{2}=1$ or $f_{0}=0$, $f_{1}=1$.

Fibonacci sequence was initiated in the book *Liber Abaci* of Fibonacci which was written in 1202. However, the sequence is based on older history. The sequence had been described earlier as Virahanka numbers in Indian mathematics [14]. In *Liber Abaci*, the sequence starts with 1, nowadays the sequence begins either with $f_{0}=0$ or with ${f_{1}=1}$.

Some of the fundamental properties of Fibonacci numbers are given as follows:

$$\begin{aligned}& \lim_{n\rightarrow\infty}\frac{f_{n+1}}{f_{n}}= \frac{1+\sqrt{5}}{2}=\alpha \quad \textrm{(golden ratio)}, \\& \sum_{k=0}^{n}f_{k}=f_{n+2}-1 \quad(n\in\mathbb{N}), \\& \sum_{k}\frac{1}{f_{k}} \textrm{ converges}, \\& f_{n-1}f_{n+1}-f_{n}^{2}=(-1)^{n+1} \quad(n\geq1) \quad\textrm{(Cassini formula)}. \end{aligned}$$

It yields $f_{n-1}^{2}+f_{n}f_{n-1}-f_{n}^{2}=(-1)^{n+1}$ if we can substitute for $f_{n+1}$ in Cassini’s formula.

Let $f_{n}$ be the *n*th Fibonacci number for every $n\in\mathbb{N}$. Then we define the infinite matrix $\widehat{F}=(\widehat{f}_{nk})$ [15] by

$$\widehat{f}_{nk}= \textstyle\begin{cases} -\frac{f_{n+1}}{f_{n}} & (k=n-1), \\ \frac{f_{n}}{f_{n+1}} & (k=n), \\ 0 & (0\leq k < n-1 \text{ or } k>n). \end{cases} $$

### Approximation theory

Korovkin type approximation theorems are practical tools to check whether a given sequence $(A_{n})_{n\geq1}$ of positive linear operators on $C[a,b]$ of all continuous functions on the real interval $[a,b]$ is an approximation process. That is, these theorems present a variety of test functions which provide that the approximation property holds on the whole space if it holds for them. Such a property was determined by Korovkin [16] in 1953 for the functions 1, *x* and $x^{2}$ in the space $C[a,b]$ as well as for the functions 1, cos and sin in the space of all continuous 2*π*-periodic functions on the real line.

Until the study of Gadjiev and Orhan [17], there was no study related to statistical convergence and approximation theory. In [17], Korovkin type approximation theorems were proved by using the idea of statistical convergence. Some of the examples of approximation theory and statistical convergence studies can be seen in [9, 10, 18–24].

## Methods

In the theory of numbers, there are many different definitions of density. It is well known that the most popular of these definitions is asymptotic density. However, asymptotic density does not exist for all sequences. New densities have been defined to fill those gaps and to serve different purposes.

The asymptotic density is one of the possibilities to measure how large a subset of the set of natural numbers is. We know intuitively that positive integers are much more than perfect squares. Because every perfect square is positive and many other positive integers exist besides. However, the set of positive integers is not in fact larger than the set of perfect squares: both sets are infinite and countable and can therefore be put in one-to-one correspondence. Nevertheless, if one goes through the natural numbers, the squares become increasingly scarce. It is precisely in this case that natural density helps us and makes this intuition precise.

The Fibonacci sequence was firstly used in the theory of sequence spaces by Kara and Başarır [25]. Afterward, Kara [15] defined the Fibonacci difference matrix *F̂* by using the Fibonacci sequence $(f_{n})$ for $n\in\{0,1,\ldots\}$ and introduced the new sequence spaces related to the matrix domain of *F̂*.

Following [25] and [15], high quality papers have been produced on the Fibonacci matrix by many mathematicians [26–36].

In this paper, by combining the definitions of Fibonacci sequence and statistical convergence, we obtain a new concept of statistical convergence, which will be called Fibonacci type statistical convergence. We examine some basic properties of new statistical convergence defined by Fibonacci sequences. Henceforth, we get an analogue of the classical Korovkin theorem by using the concept of Fibonacci type statistical convergence.

It will be shown that if *X* is a Banach space, then for a closed subset of *X*, which is denoted by *A*, Fibonacci type space *A* is closed in Fibonacci type space *X*. We will give the definitions of Fibonacci statistically Cauchy sequence and investigate the Fibonacci statistically convergent sequences and Fibonacci statistically Cauchy sequences. Using the definition of statistical boundedness, it will be proved that the set of Fibonacci statistically convergent sequence spaces of real numbers is a closed linear space of a set of Fibonacci bounded sequences of real numbers and nowhere dense in Fibonacci bounded sequences of real numbers. After proving that the set of Fibonacci statistically convergent sequences is dense in Frechet metric space of all real sequences, the inclusion relations will be given.

For the rest of the paper, firstly an approximation theorem, which is an analogue of Korovkin theorem, is given and an example is solved. Second, the rate of Fibonacci statistical convergence of a sequence of positive linear operators defined $C_{2\pi}(\mathbb{R})$ into $C_{2\pi}(\mathbb{R})$ is computed.

## Main results

### Fibonacci type statistical convergence

Now, we give the general Fibonacci sequence space $X(\widehat{F})$ as follows [15, 25]: Let *X* be any sequence space and $k\in\mathbb{N}$. Then

$$X(\widehat{F})= \bigl\{ x=(x_{k})\in\omega: ( \widehat {F}x_{k} ) \in X \bigr\} . $$

It is clear that if *X* is a linear space, then $X(\widehat{F})$ is also a linear space. Kara proved that if *X* is a Banach space, then $X(\widehat{F})$ is also a Banach space with the norm

$$\Vert x\Vert _{X(\widehat{F})}=\Vert \widehat{F}x\Vert _{X}. $$

Now, we will give lemma which is used in the proof of Theorem 3.2. Proof of this lemma is trivial.

#### Lemma 3.1

*If* $X\subset Y$, *then* $X(\widehat{F}) \subset Y(\widehat{F})$.

#### Theorem 3.2

*Consider that* *X* *is a Banach space and* *A* *is a closed subset of X*. *Then* $A(\widehat{F})$ *is also closed in* $X(\widehat{F})$.

#### Proof

Since *A* is a closed subset of *X*, from Lemma 3.1, then we can write $A(\widehat{F}) \subset X(\widehat{F})$. $\overline{A( \widehat{F})}$, *A̅* denote the closure of $A(\widehat{F})$ and *A*, respectively. To prove the theorem, we must show that $\overline{A(\widehat{F})}=\overline{A}(\widehat{F})$.

Firstly, we take $x\in\overline{A(\widehat{F})}$. Therefore, from 1.4.6 Theorem of [37], there exists a sequence $(x^{n}) \in A( \widehat{F})$ such that $\Vert x^{n}-x\Vert _{\widehat {F}}\rightarrow0$ in $A(\widehat{F})$ for $n\rightarrow\infty$. Thus, $\Vert (x^{n}_{k})-(x _{k})\Vert _{\widehat{F}}\rightarrow0$ as $n\rightarrow\infty$ in $A(\widehat{F})$ so that

$$\sum_{i=1}^{m}\bigl\vert x_{i}^{n}-x_{i}\bigr\vert +\bigl\Vert \widehat{F}\bigl(x_{k} ^{n}\bigr)-\widehat{F}(x_{k}) \bigr\Vert \rightarrow0 $$

for $n\rightarrow\infty$, in *A*. Therefore, $\widehat{F}x \in \overline{A}$ and so $x\in\overline{A}(\widehat{F})$.

Conversely, if we take $x\in\overline{A(\widehat{F})}$, then $x\in A(\widehat{F})$. We know that *A* is closed. Then $\overline{A( \widehat{F})}=\overline{A}(\widehat{F})$. Hence, $A(\widehat{F})$ is a closed subset of $X(\widehat{F})$. □

From this theorem, we can give the following corollary.

#### Corollary 3.3

*If* *X* *is a separable space*, *then* $X(\widehat{F})$ *is also a separable space*.

#### Definition 3.4

A sequence $x=(x_{k})$ is said to be Fibonacci statistically convergent (or *F̂*-statistically convergent) if there is a number *L* such that, for every $\epsilon> 0$, the set $K_{\epsilon}( \widehat{F}):=\{k\leq n:\vert \widehat{F}x_{k}-L\vert \geq \epsilon\}$ has natural density zero, i.e., $d(K_{\epsilon}(\widehat{F}))=0$. That is,

$$\lim_{n \to\infty}\frac{1}{n}\bigl\vert \bigl\{ k\leq n: \vert \widehat{F}x_{k}-L\vert \geq\epsilon\bigr\} \bigr\vert =0. $$

In this case we write $d(\widehat{F})-\lim x_{k}=L$ or $x_{k}\rightarrow L(S(\widehat{F}))$. The set of *F̂*-statistically convergent sequences will be denoted by $S(\widehat{F})$. In the case $L=0$, we will write $S_{0}(\widehat{F})$.

#### Definition 3.5

Let $x=(x_{k})\in\omega$. The sequence *x* is said to be *F̂*-statistically Cauchy if there exists a number $N=N( \varepsilon)$ such that

$$\lim_{n \to\infty}\frac{1}{n}\bigl\vert \bigl\{ k\leq n: \vert \widehat{F}x_{k}- \widehat{F}x_{N}\vert \geq \epsilon\bigr\} \bigr\vert =0 $$

for every $\varepsilon>0$.

#### Theorem 3.6

*If* *x* *is an* *F̂*-*statistically convergent sequence*, *then* *x* *is an* *F̂*-*statistically Cauchy sequence*.

#### Proof

Let $\varepsilon>0$. Assume that $x_{k}\rightarrow L(S(\widehat{F}))$. Then $\vert \widehat{F}x_{k}-L\vert <\varepsilon/ 2$ for almost all *k*. If *N* is chosen so that $\vert \widehat{F}x_{N}-L\vert <\varepsilon/ 2$, then we have $\vert \widehat{F}x_{k}-\widehat{F}x_{N}\vert < \vert \widehat{F}x_{k}-L\vert +\vert \widehat{F}x_{N}-L\vert <\varepsilon/ 2 + \varepsilon/ 2 =\varepsilon$ for almost all *k*. It means that *x* is an *F̂*-statistically Cauchy sequence. □

#### Theorem 3.7

*If* *x* *is a sequence for which there is an* *F̂*-*statistically convergent sequence* *y* *such that* $\widehat{F}x_{k}=\widehat{F}y_{k}$ *for almost all* *k*, *then* *x* *is an* *F̂*- *statistically convergent sequence*.

#### Proof

Suppose that $\widehat{F}x_{k}=\widehat{F}y_{k}$ for almost all *k* and $y_{k}\rightarrow L(S(\widehat{F}))$. Then $\varepsilon>0$ and, for each *n*, $\{k \leq n: \vert \widehat{F}x_{k}-L\vert \geq \varepsilon\}\subseteq \{k \leq n: \widehat{F}x_{k}\neq\widehat{F}y_{k}\} \cup\{k \leq n: \vert \widehat{F}x_{k}-L\vert \leq\varepsilon\}$. Since $y_{k}\rightarrow L(S( \widehat{F}))$, the latter set contains a fixed number of integers, say $g=g(\varepsilon)$. Therefore, for $\widehat{F}x_{k}=\widehat{F}y _{k}$, for almost all *k*,

$$\lim_{n}\frac{1}{n}\bigl\vert \bigl\{ k\leq n: \vert \widehat {F}x_{k}-L\vert \geq\varepsilon \bigr\} \bigr\vert \leq\lim_{n}\frac{1}{n}\bigl\vert \{k\leq n: \widehat{F}x_{k} \neq\widehat{F}y_{k} \}\bigr\vert +\lim _{n}\frac{g}{n}=0. $$

Hence $x_{k}\rightarrow L(S(\widehat{F}))$. □

#### Definition 3.8

[38]

A sequence $x=(x_{k})$ is said to be statistically bounded if there exists some $L\geq0$ such that

$$d \bigl( \bigl\{ k: \vert x_{k}\vert >L\bigr\} \bigr) =0, \quad \textrm {i.e.,} \quad \vert x_{k}\vert \leq L \quad \textrm{a.a.k.} $$

By $m_{0}$, we denote the linear space of all statistically bounded sequences. Bounded sequences are obviously statistically bounded as the empty set has zero natural density. However, the converse is not true. For example, we consider the sequence

$$x_{n} = \textstyle\begin{cases} n& (k\text{ is a square}), \\ 0& (k\text{ is not a square}). \end{cases} $$

Clearly $(x_{k})$ is not a bounded sequence. However, $d(\{k: \vert x_{k}\vert >1/2 \})=0$, as the set of squares has zero natural density and hence $(x_{k})$ is statistically bounded [39].

#### Proposition 3.9

[39]

*Every convergent sequence is statistically bounded*.

Although a statistically convergent sequence does not need to be bounded (cf. [39, 40]), the following proposition shows that every statistically convergent sequence is statistically bounded.

#### Proposition 3.10

[39]

*Every statistically convergent sequence is statistically bounded*.

Now, using Propositions 3.9 and 3.10, we can give the following corollary.

#### Corollary 3.11

*Every* *F̂*-*statistically convergent sequence is* *F̂*-*statistically bounded*.

Denote the set of all *F̂*-bounded sequences of real numbers by $m(\widehat{F})$ [15]. Based on Definition 3.8 and the descriptions of $m_{0}$ and $m(\widehat{F})$, we can denote the set of all *F̂*-bounded statistically convergent sequences of real numbers by $m_{0}(\widehat{F})$.

The following theorem can be proved by Theorem 2.1 of [41] and Theorem 3.2.

#### Theorem 3.12

*The set of* $m_{0}(\widehat{F})$ *is a closed linear space of the linear normed space* $m(\widehat{F})$.

#### Theorem 3.13

*The set of* $m_{0}(\widehat{F})$ *is a nowhere dense set in* $m( \widehat{F})$.

#### Proof

According to [41], every closed linear subspace of an arbitrary linear normed space *E*, different from *E*, is a nowhere dense set in *E*. Hence, on account of Theorem 3.12, it suffices to prove that $m_{0}(\widehat{F})\neq m(\widehat{F})$. But this is evident, consider the sequence

$$x_{n} = \textstyle\begin{cases} 1& (n\text{ is odd}), \\ 0& (n\text{ is even}). \end{cases} $$

Then $x\in m(\widehat{F})$, but does not belong to $m_{0}(\widehat{F})$. □

*ω* denotes the Fréchet metric space of all real sequences with the metric $d_{\omega}$,

$$d_{\omega}=\sum_{k=1}^{\infty} \frac{1}{2^{k}}\frac{\vert x_{k}-y_{k}\vert }{1+\vert x _{k}-y_{k}\vert }, $$

where $x=(x_{k}), y=(y_{k})\in\omega$ for all $k=1,2,\ldots$ .

#### Theorem 3.14

*The set of* *F̂*-*statistically convergent sequences is dense in the space ω*.

#### Proof

If $x=(x_{k})\in S(\widehat{F})$ (for all *k*) and the sequence $y=(y_{k})$ (for all *k*) of real numbers differs from *x* only in a finite number of terms, then evidently $y\in S(\widehat{F})$, too. From this statement the proof follows at once on the basis of the definition of the metric in *ω*. □

#### Theorem 3.15

*The following statements hold*.

i. *The inclusion* $c(\widehat{F})\subset S(\widehat{F})$ *is strict*.
ii. $S(\widehat{F})$ *and* $\ell_{\infty}(\widehat{F})$ *overlap but neither one contains the other*.
iii. $S(\widehat{F})$ *and* $\ell_{\infty}$ *overlap but neither one contains the other*.
iv. *S* *and* $S(\widehat{F})$ *overlap but neither one contains the other*.
v. *S* *and* $c(\widehat{F})$ *overlap but neither one contains the other*.
vi. *S* *and* $c_{0}(\widehat{F})$ *overlap but neither one contains the other*.
vii. *S* *and* $\ell_{\infty}(\widehat{F})$ *overlap but neither one contains the other*.

#### Proof

i) Since $c\subset S$, then $c(\widehat{F})\subset S(\widehat{F})$. We choose

3.1 $$\begin{aligned} \widehat{F}x_{n}=\bigl(f_{n+1}^{2} \bigr)=\bigl(1,2^{2},3^{2},5^{2},\ldots\bigr). \end{aligned}$$

Since $f_{n+1}^{2}\rightarrow\infty$ as $k\rightarrow\infty$ and $\widehat{F}x=(1,0,0,\ldots)$, then $\widehat{F}x\in S$, but is not in the space *c*, that is, $\widehat{F} \notin c$.

For the other items, firstly, use the inclusion relations in [15]. It is obtained that the inclusions $c\subset S( \widehat{F})$, $c\subset c(\widehat{F})$, $c\subset m(\widehat{F})$, $c\subset S$, $c\subset\ell_{\infty}$ and $c\cap c_{0}(\widehat{F}) \neq\phi$ hold. Then we see that $S(\widehat{F})$ and $\ell_{\infty }(\widehat{F})$, $S(\widehat{F})$ and $\ell_{\infty}$, *S* and $S(\widehat{F})$, *S* and $c(\widehat{F})$, *S* and $c_{0}( \widehat{F})$, *S* and $\ell_{\infty}(\widehat{F})$ overlap.

ii) We define $\widehat{F}x=\widehat{F}x_{n}$ by (3.1). Then $\widehat{F}x\in S$, but *F̂x* is not in $\ell_{\infty}$. Now we choose $u=(1,0,1,0,\ldots)$. Then $u\in\ell_{\infty}( \widehat{F})$ but $u\notin S(\widehat{F})$.

iii) The proof is the same as (ii).

iv) Define

$$x_{n} = \textstyle\begin{cases} 1 & (n\text{ is a square}), \\ 0 & (\text{otherwise}). \end{cases} $$

Then $x\in S$, but $x \notin S(\widehat{F})$. Conversely, if we take $u=(n)$, then $u\notin S$ but $x \in S(\widehat{F})$.

(v), (vi) and (vii) are proven similar to (iv). □

### Approximation theorems

#### Approximation by *F̂*-statistical convergence

In this section, we get an analogue of classical Korovkin theorem by using the concept of *F̂*-statistical convergence.

Let $F(\mathbb{R})$ denote the linear space of real-valued functions on $\mathbb{R}$. Let $C(\mathbb{R})$ be a space of all real-valued continuous functions *f* on $\mathbb{R}$. It is well known that $C(\mathbb{R})$ is a Banach space with the norm given as follows:

$$\Vert f\Vert _{\infty}=\sup_{x\in\mathbb{R}}\bigl\vert f(x) \bigr\vert ,\quad f\in C(\mathbb{R}), $$

and we denote by $C_{2\pi}(\mathbb{R})$ the space of all 2*π*-periodic functions $f\in C(\mathbb{R})$, which is a Banach space with the norm given by

$$\Vert f\Vert _{2\pi}=\sup_{t\in\mathbb{R}}\bigl\vert f(t) \bigr\vert ,\quad f\in C(\mathbb{R}). $$

We say *A* is a positive operator if for every non-negative *f* and $x\in I$, we have $A(f,x)\geq0$, where *I* is any given interval on the real semi-axis. The first and second classical Korovkin approximation theorems state the following (see [16, 42]).

##### Theorem 3.16

*Let* $(A_{n})$ *be a sequence of positive linear operators from* $C[0,1]$ *into* $F[0,1]$. *Then*

$$\lim_{n \to\infty}\bigl\Vert A_{n}(f,x)-f(x)\bigr\Vert _{C[a,b]}=0 \quad \Leftrightarrow\quad \lim_{n \to\infty}\bigl\Vert A_{n}(e_{i}, x)-e_{i}\bigr\Vert _{C[a,b]}=0, $$

*where* $e_{i}=x^{i}$, $i=0,1,2$.

##### Theorem 3.17

*Let* $(T_{n})$ *be a sequence of positive linear operators from* $C_{2\pi}(\mathbb{R})$ *into* $F(\mathbb{R})$. *Then*

$$\lim_{n \to\infty}\bigl\Vert T_{n}(f,x)-f(x)\bigr\Vert _{2\pi}=0 \quad \Leftrightarrow\quad \lim_{n \to\infty}\bigl\Vert T_{n}(f_{i}, x)-f_{i}\bigr\Vert _{2\pi}=0, \quad i=0,1,2, $$

*where* $f_{0}=1$, $f_{1}=sinx$ *and* $f_{2}=cosx$.

Our main Korovkin type theorem is given as follows.

##### Theorem 3.18

*Let* $(L_{k})$ *be a sequence of positive linear operators from* $C_{2\pi}(\mathbb{R})$ *into* $C_{2\pi}(\mathbb{R})$. *Then*, *for all* $f\in C_{2\pi}(\mathbb{R})$,

3.2 $$\begin{aligned} d(\widehat{F})-\lim_{k \to\infty}\bigl\Vert L_{k}(f,x)-f(x) \bigr\Vert _{2\pi}=0 \end{aligned}$$

*if and only if*

3.3 $$\begin{aligned}& d(\widehat{F})-\lim_{k \to\infty}\bigl\Vert L_{k}(1,x)-1 \bigr\Vert _{2\pi}=0 , \end{aligned}$$

3.4 $$\begin{aligned}& d(\widehat{F})-\lim_{k \to\infty}\bigl\Vert L_{k}(\sin t,x)-\sin x \bigr\Vert _{2\pi}=0, \end{aligned}$$

3.5 $$\begin{aligned}& d(\widehat{F})-\lim_{k \to\infty}\bigl\Vert L_{k}(\cos t,x)-\cos x \bigr\Vert _{2\pi}=0. \end{aligned}$$

##### Proof

As $1,\sin x,\cos x\in C_{2\pi}(\mathbb{R})$, conditions (3.3)-(3.5) follow immediately from (3.2). Let conditions (3.3)-(3.5) hold and $I_{1}=(a,a+2\pi)$ be any subinterval of length 2*π* in $\mathbb{R}$. Let us fix $x\in I_{1}$. By the properties of function *f*, it follows that for given $\varepsilon> 0$ there exists $\delta=\delta(\epsilon)>0$ such that

3.6 $$ \bigl\vert f(x)-f(t)\bigr\vert < \varepsilon, \quad \textrm{whenever } \forall \vert t-x\vert < \delta. $$

If $\vert t-x\vert \geq\delta$, let us assume that $t\in (x+\delta,2\pi+x+ \delta)$. Then we obtain that

3.7 $$ \bigl\vert f(x)-f(t)\bigr\vert \leq2\Vert f\Vert _{2\pi }\leq\frac{2\Vert f \Vert _{2\pi}}{\sin^{2} ( \frac{\delta}{2} ) }\psi(t), $$

where $\psi(t)=\sin^{2} ( \frac{t-x}{2} ) $.

By using (3.6) and (3.7), we have

$$\bigl\vert f(x)-f(t)\bigr\vert < \varepsilon+ \frac{2\Vert f\Vert _{2\pi}}{\sin ^{2} ( \frac{\delta}{2} ) }\psi(t). $$

This implies that

$$-\varepsilon-\frac{2\Vert f\Vert _{2\pi}}{\sin^{2} ( \frac{ \delta}{2} ) }\psi(t)< f(x)-f(t)< \varepsilon+ \frac{2\Vert f\Vert _{2\pi}}{\sin^{2} ( \frac{\delta}{2} ) } \psi(t). $$

By using the positivity and linearity of $\{L_{k}\}$, we get

$$L_{k}(1,x) \biggl( -\varepsilon\frac{2\Vert f\Vert _{2\pi }}{\sin ^{2} ( \frac{\delta}{2} ) }\psi(t) \biggr) < L_{k}(1,x) \bigl( f(x)-f(t) \bigr) < L_{k}(1,x) \biggl( \varepsilon+ \frac{2\Vert f\Vert _{2\pi}}{ \sin^{2} ( \frac{\delta}{2} ) }\psi(t) \biggr) , $$

where *x* is fixed and so $f(x)$ is a constant number. Therefore,

3.8 $$\begin{aligned} -\varepsilon L_{k}(1,x)-\frac{2\Vert f\Vert _{2\pi}}{\sin^{2} ( \frac{\delta}{2} ) }L_{k} \bigl(\psi(t),x\bigr) < & L_{k}(f,x)-f(x)L _{k}(1,x) \\ < &\varepsilon L_{k}(1,x)+\frac{2\Vert f\Vert _{2\pi}}{\sin^{2} ( \frac{\delta}{2} ) }L_{k}\bigl( \psi(t),x\bigr). \end{aligned}$$

On the other hand, we get

3.9 $$\begin{aligned} L_{k}(f,x)-f(x) =&L_{k}(f,x)-f(x)L_{k}(1,x)+f(x)L_{k}(1,x)-f(x) \\ =&L_{k}(f,x)-f(x)L_{k}(1,x)-f(x)L_{k}+f(x) \bigl[L_{k}(1,x)-1\bigr] . \end{aligned}$$

By inequalities (3.8) and (3.9), we obtain

3.10 $$\begin{aligned} L_{k}(f,x)-f(x) < &\varepsilon L_{k}(1,x)+ \frac{2\Vert f \Vert _{2\pi}}{\sin^{2} ( \frac{\delta}{2} ) }L_{k}\bigl( \psi(t),x\bigr) \\ &{}+f(x)+f(x)\bigl[L_{k}(1,x)-1\bigr]. \end{aligned}$$

Now, we compute the second moment

$$\begin{aligned} L_{k}\bigl(\psi(t),x\bigr) =&L_{k} \biggl( \sin^{2} \biggl( \frac{x-t}{2} \biggr) ,x \biggr) =L _{k} \biggl( \frac{1}{2}(1-\cos t \cos x-\sin x \sin t),x \biggr) \\ =&\frac{1}{2}\bigl[L_{k}(1,x)-\cos xL_{k}(\cos t,x)- \sin xL_{k}(\sin t ,x)\bigr] \\ =&\frac{1}{2}\bigl\{ L_{k}(1,x)-\cos x\bigl[L_{k}( \cos t,x)-\cos x\bigr] \\ &{} -\sin x\bigl[L_{k}(\sin t ,x)-\sin x\bigr]\bigr\} . \end{aligned}$$

By (3.10), we have

$$\begin{aligned} L_{k}(f,x)-f(x) < &\varepsilon L_{k}(1,x)+ \frac{2\Vert f \Vert _{2\pi}}{\sin^{2} ( \frac{\delta}{2} ) }\frac{1}{2} \bigl\{ L_{k}(1,x) \\ &{}-\cos x\bigl[L_{k}(\cos t,x)-\cos x\bigr]-\sin x \bigl[L_{k}(\sin t ,x)-\sin x\bigr]\bigr\} \\ &{} +f(x) \bigl(L _{k}(1,x)-1\bigr) \\ =&\varepsilon\bigl[L_{k}(1,x)-1\bigr]+\varepsilon+f(x) \bigl(L_{k}(1,x)-1\bigr) \\ &{}+ \frac{\Vert f\Vert _{2\pi}}{\sin^{2} ( \frac{\delta }{2} ) } \bigl\{ L_{k}(1,x)-\cos x\bigl[L_{k}( \cos t,x)-\cos x\bigr] \\ &{}-\sin x\bigl[L_{k}(\sin t ,x)-\sin x\bigr]\bigr\} . \end{aligned}$$

So, from the above inequality, one can see that

$$\begin{aligned} \bigl\vert L_{k}(f,x)-f(x)\bigr\vert & \leq \varepsilon+ \biggl( \varepsilon+\bigl\vert f(x)\bigr\vert +\frac{ \Vert f\Vert _{2\pi}}{\sin^{2} ( \frac{\delta }{2} ) } \biggr)\bigl\vert L _{k}(1,x)-1\bigr\vert \\ & \quad {}+ \frac{\Vert f\Vert _{2\pi}}{\sin^{2} ( \frac{\delta }{2} ) }\bigl[\vert \cos x\vert \bigl\vert L_{k}(\cos t,x)-\cos x\bigr\vert \\ &\quad {}+\vert \sin x\vert \big\vert L_{k}(\sin t,x)-\sin x \big\vert \bigr] \\ &\leq \varepsilon+ \biggl( \varepsilon +\bigl\vert f(x)\bigr\vert + \frac{\Vert f\Vert _{2\pi }}{\sin^{2} ( \frac{ \delta}{2} ) } \biggr) \bigl\vert L_{k}(1,x)-1\bigr\vert \\ & \quad {}+ \frac{\Vert f\Vert _{2\pi}}{\sin^{2} ( \frac{\delta }{2} ) }\bigl[\bigl\vert L _{k}(\cos t,x)- \cos x\bigr\vert +\vert \sin x\vert \big\vert L_{k}(\sin t ,x)-\sin x \big\vert \bigr]. \end{aligned}$$

Because *ε* is arbitrary, we obtain

$$\begin{aligned} \bigl\Vert L_{k}(f,x)-f(x)\bigr\Vert _{2\pi} \leq& \varepsilon+R \bigl(\bigl\Vert L_{k}(1,x)-1\bigr\Vert _{2\pi}+\bigl\Vert L_{k}(\cos t,x)- \cos t\bigr\Vert _{2\pi} \\ &{}+\bigl\Vert L_{k}(\sin t,x)-\sin x\bigr\Vert _{2\pi} \bigr), \end{aligned}$$

where $R=\max ( \varepsilon+\Vert f\Vert _{2\pi }+\frac{ \Vert f\Vert _{2\pi}}{\sin^{2} ( \frac{\delta }{2} ) },\frac{ \Vert f\Vert _{2\pi}}{\sin^{2} ( \frac{\delta }{2} ) } ) $.

Finally, replacing $L_{k}(\cdot,x)$ by $T_{k}(\cdot,x)=\widehat{F}L_{k}(\cdot,x)$ and for $\varepsilon^{\prime}>0$, we can write

$$\begin{aligned}& \begin{aligned} \mathcal{A}: ={}& \biggl\{ k \in\mathbb{N}: \\ &{}\bigl\Vert T_{k}(1,x)-1 \bigr\Vert _{2\pi}+\bigl\Vert T_{k}(\sin t,x)-\sin x\bigr\Vert _{2\pi}+ \bigl\Vert T_{k}(\cos t,x)-\cos x\bigr\Vert _{2\pi}\geq\frac{ \varepsilon^{\prime}}{R} \biggr\} , \end{aligned} \\& \mathcal{A}_{1}: = \biggl\{ k \in\mathbb{N}:\bigl\Vert T_{k}(1,x)-1 \bigr\Vert _{2\pi}\geq\frac{\varepsilon^{\prime}}{3R} \biggr\} , \\& \mathcal{A}_{2}: = \biggl\{ k \in\mathbb{N}:\bigl\Vert T_{k}(\sin t,x)- \sin x\bigr\Vert _{2\pi}\geq \frac{\varepsilon^{\prime}}{3R} \biggr\} , \\& \mathcal{A}_{3}: = \biggl\{ k \in\mathbb{N}:\bigl\Vert T_{k}(\cos t,x)- \cos x\bigr\Vert _{2\pi}\geq \frac{\varepsilon^{\prime}}{3R} \biggr\} . \end{aligned}$$

Then $\mathcal{A}\subset\mathcal{A}_{1}\cup\mathcal{A}_{2}\cup \mathcal{A}_{3}$, so we have $d(\mathcal{A})\leq d(\mathcal{A}_{1})+d( \mathcal{A}_{2})+d(\mathcal{A}_{3})$. Thus, by conditions (3.3)-(3.5), we obtain

$$d(\widehat{F})-\lim_{k \to\infty}\bigl\Vert L_{k}(f,x)-f(x) \bigr\Vert _{2\pi}=0, $$

which completes the proof. □

We remark that our Theorem 3.18 is stronger than Theorem 3.17 as well as Theorem of Gadjiev and Orhan [17]. For this purpose, we get the following example.

##### Example 3.19

For $n\in\mathbb{N}$, denote by $S_{n}(f)$ the *n*-partial sum of the Fourier series of *f*, that is,

$$S_{n}(f,x)=\frac{1}{2}a_{0}(f)+\sum _{k=0}^{n}a_{k}(f)\cos kx+b_{k}(f) \sin k x. $$

For $n\in\mathbb{N}$, we get

$$F_{n}(f,x)=\frac{1}{n+1}\sum_{k=0}^{n}S_{k}(f). $$

A standard calculation gives that for every $t\in\mathbb{R}$

$$F_{n}(f,x)=\frac{1}{2\pi} \int_{-\pi}^{\pi}f(t)\varphi_{n}(x-t)dt, $$

where

$$\varphi_{n}(x) = \textstyle\begin{cases} \frac{\sin^{2}((n+1)(x-t)/2)}{(n+1)\sin^{2}((x-t)/2)},& \text{if } x\text{ is not a multiple of }2\pi, \\ n+1, & \text{if }x\text{ is a multiple of }2\pi. \end{cases} $$

The sequence $(\varphi_{n})_{n\in\mathbb{N}}$ is a positive kernel which is called the Fejér kernel, and corresponding $F_{n}$ for $n\geq1$ are called Fejér convolution operators.

We define the sequence of linear operators as $K_{n}:C_{2\pi}( \mathbb{R})\longrightarrow C_{2\pi}(\mathbb{R})$ with $K_{n}(f,x)=(1+y _{n})F_{n}(f,x)$, where $y=(y_{n})=(f^{2}_{n+1})$. Then $K_{n}(1,x)=1$, $K_{n}(\sin t,x)=\frac{n}{n+1}\sin x$ and $K_{n}(\cos t,x)= \frac{n}{n+1}\cos x$ and the sequence $(K_{n})$ satisfies conditions (3.3)-(3.5). Therefore, we get

$$d(\widehat{F})-\lim_{k \to\infty}\bigl\Vert K_{n}(f,x)-f(x) \bigr\Vert _{2\pi}=0. $$

On the other hand, one can see that $(K_{n})$ does not satisfy Theorem 3.17 as well as Theorem of Gadjiev and Orhan [17] since $\widehat{F}y=(1,0,0,\ldots)$, the sequence *y* is *F̂*-statistically convergent to 0. But the sequence *y* is neither convergent nor statistically convergent.

#### Rate of *F̂*-statistical convergence

In this section, we estimate the rate of *F̂*-statistical convergence of a sequence of positive linear operators defined by $C_{2\pi}(\mathbb{R})$ into $C_{2\pi}(\mathbb{R})$. Now, we give the following definition.

##### Definition 3.20

Let $(a_{n})$ be a positive non-increasing sequence. We say that the sequence $x=(x_{k})$ is *F̂*-statistically convergent to *ℓ* with the rate $o(a_{n})$ if, for every $\varepsilon>0$,

$$\lim_{n \to\infty}\frac{1}{u_{n}}\bigl\vert \bigl\{ k\leq n: \vert \widehat{F}x- \ell \vert \geq\epsilon \bigr\} \bigr\vert =0. $$

At this stage, we can write $x_{k}-\ell=d(\widehat{F})-o(u_{n})$.

As usual we have the following auxiliary result.

##### Lemma 3.21

*Let* $(a_{n})$ *and* $(b_{n})$ *be two positive non*-*increasing sequences*. *Let* $x=(x_{k})$ *and* $y=(y_{k})$ *be two sequences such that* $x_{k}-L _{1}=d(\widehat{F})-o(a_{n})$ *and* $y_{k}-L_{1}=d(\widehat{F})-o(b_{n})$. *Then we have*

(i) $\alpha(x_{k}-L_{1})=d(\widehat{F})-o(a_{n})$ *for any scalar* *α*,
(ii) $(x_{k}-L_{1})\pm(y_{k}-L_{2})=d(\widehat{F})-o(c_{n})$,
(iii) $(x_{k}-L_{1})(y_{k}-L_{2})=d(\widehat{F})-o(a_{n}b_{n})$,

*where* $c_{n}= \max\{a_{n},b_{n}\}$.

For $\delta> 0$, the modulus of continuity of *f*, $\omega(f,\delta )$ is defined by

$$ \omega(f,\delta)=\sup_{\vert x-y\vert < \delta}\bigl\vert f(x)-f(y)\bigr\vert . $$

It is well known that for a function $f \in C[a,b]$,

$$ \lim_{n\to0^{+}}\omega(f,\delta)=0 $$

for any $\delta>0$

3.11 $$ \bigl\vert f(x)-f(y)\bigr\vert \leq\omega(f,\delta) \biggl( \frac {\vert x-y\vert }{\delta}+1 \biggr) . $$

##### Theorem 3.22

*Let* $(L_{k})$ *be a sequence of positive linear operators from* $C_{2\pi}(\mathbb{R})$ *into* $C_{2\pi}(\mathbb{R})$. *Assume that*

$$\begin{aligned}& \textup{(i)}\quad \bigl\Vert L_{k}(1,x)-x\bigr\Vert _{2\pi}=d(\widehat{F})-o(u_{n}), \\& \textup{(ii)}\quad \omega(f,\theta_{k})=d(\widehat{F})-o(v_{n}), \quad \textit{where } \theta_{k}= \sqrt{L_{k} \biggl[ \sin^{2} \biggl( \frac{t-x}{2} \biggr) ,x \biggr] }. \end{aligned}$$

*Then*, *for all* $f\in C_{2\pi}(\mathbb{R})$, *we get*

$$\bigl\Vert L_{k}(f,x)-f(x)\bigr\Vert _{2\pi}=d( \widehat{F})-o(z_{n}), $$

*where* $z_{n}= \max\{u_{n},v_{n}\}$.

##### Proof

Let $f\in C_{2\pi}(\mathbb{R})$ and $x\in[-\pi,\pi]$. From (3.9) and (3.11), we can write

$$\begin{aligned} \bigl\vert L_{k}(f,x)-f(x)\bigr\vert \leq&L_{k}\bigl( \bigl\vert f(t)-f(x)\bigr\vert ;x\bigr)+\bigl\vert f(x)\bigr\vert \bigl\vert L_{k}(1,x)-1\bigr\vert \\ \leq&L_{k} \biggl( \frac{\vert x-y\vert }{\delta}+1;x \biggr) \omega(f,\delta)+ \bigl\vert f(x)\bigr\vert \bigl\vert L _{k}(1,x)-1\bigr\vert \\ \leq&L_{k} \biggl( \frac{\pi^{2}}{\delta^{2}}\sin^{2} \biggl( \frac {y-x}{2} \biggr) +1;x \biggr) \omega(f,\delta)+\bigl\vert f(x)\bigr\vert \bigl\vert L_{k}(1,x)-1\bigr\vert \\ \leq& \biggl\{ L_{k}(1,x)+\frac{\pi^{2}}{\delta^{2}}L_{k} \biggl( \sin ^{2} \biggl( \frac{y-x}{2} \biggr) ;x \biggr) \biggr\} \omega(f,\delta )+\bigl\vert f(x)\bigr\vert \bigl\vert L _{k}(1,x)-1\bigr\vert \\ =& \biggl\{ L_{k}(1,x)+\frac{\pi^{2}}{\delta^{2}}L_{k} \biggl( \sin^{2} \biggl( \frac{y-x}{2} \biggr) ;x \biggr) \biggr\} \omega(f,\delta)+\bigl\vert f(x)\bigr\vert \bigl\vert L _{k}(1,x)-1 \bigr\vert . \end{aligned}$$

By choosing $\sqrt{\theta_{k}}=\delta$, we get

$$\begin{aligned} \bigl\Vert L_{k}(f,x)-f(x)\bigr\Vert _{2\pi} \leq& \Vert f \Vert _{2\pi}\bigl\Vert L_{k}(1,x)-x \bigr\Vert _{2\pi }+2\omega(f, \theta_{k})+\omega(f,\theta_{k}) \bigl\Vert L_{k}(1,x)-x \bigr\Vert _{2 \pi} \\ \leq&K\bigl\{ \bigl\Vert L_{k}(1,x)-x \bigr\Vert _{2\pi}+ \omega(f,\theta _{k})+\omega(f,\theta_{k})\bigl\Vert L_{k}(1,x)-x \bigr\Vert _{2\pi}\bigr\} , \end{aligned}$$

where $K=\max\{2,\Vert f\Vert _{2\pi}\}$. By Definition 3.20 and conditions (i) and (ii), we get the desired result. □

## Conclusion

One of the most known and interesting number sequences is the Fibonacci sequence, and it still continues to be of interest to mathematicians because this sequence is an important and useful tool to expand the mathematical horizon for many mathematicians.

The concept of statistical convergence for a sequence of real numbers was defined by Fast [2] and Steinhaus [43] independently in 1951. Statistical convergence has recently become an area of active research. Currently, researchers in statistical convergence have devoted their effort to statistical approximation.

Approximation theory has important applications in the theory of polynomial approximation in various areas of functional analysis. The study of the Korovkin type approximation theory is a well-established area of research, which is concerned with the problem of approximating a function *f* by means of a sequence $A_{n}$ of positive linear operators. Statistical convergence is quite effective in the approximation theory. In recent times, very high quality publications have been made using approximation theory and statistical convergence [9, 10, 18–24].

In this study, we have studied the concept of statistical convergence which has an important place in the literature using Fibonacci sequences. The statistical convergence is a generalization of the usual notion of convergence. We have defined the Fibonacci type statistical convergence and investigated basic properties. A new version of Korovkin type approximation theory was introduced using the new concept of statistical convergence.
